# MBA: a literature mining system for extracting biomedical abbreviations

**DOI:** 10.1186/1471-2105-10-14

**Published:** 2009-01-09

**Authors:** Yun Xu, ZhiHao Wang, YiMing Lei, YuZhong Zhao, Yu Xue

**Affiliations:** 1 Department of Computer Science and Technology, University of Science and Technology of China Hefei, Anhui 230027, PR China; 2 Anhui Province-MOST Co-Key Laboratory of High Performance Computing and Its Application Hefei, Anhui 230027, PR China; 3 School of Life Science, University of Science and Technology of China Hefei, Anhui 230027, PR China

## Abstract

**Background:**

The exploding growth of the biomedical literature presents many challenges for biological researchers. One such challenge is from the use of a great deal of abbreviations. Extracting abbreviations and their definitions accurately is very helpful to biologists and also facilitates biomedical text analysis. Existing approaches fall into four broad categories: rule based, machine learning based, text alignment based and statistically based. State of the art methods either focus exclusively on acronym-type abbreviations, or could not recognize rare abbreviations. We propose a systematic method to extract abbreviations effectively. At first a scoring method is used to classify the abbreviations into acronym-type and non-acronym-type abbreviations, and then their corresponding definitions are identified by two different methods: text alignment algorithm for the former, statistical method for the latter.

**Results:**

A literature mining system MBA was constructed to extract both acronym-type and non-acronym-type abbreviations. An abbreviation-tagged literature corpus, called Medstract gold standard corpus, was used to evaluate the system. MBA achieved a recall of 88% at the precision of 91% on the Medstract gold-standard EVALUATION Corpus.

**Conclusion:**

We present a new literature mining system MBA for extracting biomedical abbreviations. Our evaluation demonstrates that the MBA system performs better than the others. It can identify the definition of not only acronym-type abbreviations including a little irregular acronym-type abbreviations (e.g., <CNS1, cyclophilin seven suppressor>), but also non-acronym-type abbreviations (e.g., <Fas, CD95>).

## Background

The volume of published biomedical papers is expanding at an increasing rate each year. It is very challenging for biologists to keep up to date with their own field of biomedical research with biomedical knowledge expanding so quickly. Thus, an automatic method for biomedical knowledge text mining is urgently needed [1,2]. In biomedical text mining, one special issue is the exploding use of new abbreviations [3]. It would be a great help for literature retrieval to collect these abbreviations automatically. Furthermore, other text mining tasks could be done more efficiently if all the abbreviations for an entity could be mapped to a single term representing the concept [2]. Generally, an abbreviation is a short form of a word or phrase called "definition" or "long form". Our task is to identify <"short form", "long form"> pairs where there exists a mapping from characters in the short form to characters in the long form [4].

Existing approaches fall into four broad categories: rule based, machine learning based, text alignment based, and statistically based. Rule based approaches attempt to use the best recognition rule, and good rules would result in good results. Pustejovsky et al. [4] presented a regular expression algorithm based on hand-built regular expressions, and syntactic information was considered to identify boundaries of noun phrases. Ao and Takagi [5] constructed a system called ALICE based on heuristic pattern-matching rules. Larkey et al. [6], Yu et al. [7], Park and Byrd [8] all put forward their own pattern matching rules separately. The shortcoming for these rule based approaches is that the performance of them is determined by the completeness of the rules.

Machine learning based approaches generally comprise of a learner and a predictor, and fit in with all kinds of biomedical text by learning. Chang et al. [9] presented a method for identifying abbreviations using supervised machine learning. First step they used the Longest Common Subsequence (LCS) algorithm to find all possible alignments between the definition and the abbreviation; Second step, used all the possible alignments to compute feature vectors for correctly identified definitions; Third step, used binary logistic regression to train a classifier with the feature vectors. Generally speaking, machine learning based approaches depend on the learning model and the training data, and require a lot of labor and time. Text alignment based approaches always try to find the optimal alignment between the definition and abbreviation by character matching, and are robust enough to acronym-type abbreviations. Schwartz and Hearst [10] presented a simple algorithm for identifying the definitions of abbreviations with only two indices, lIndex for the long form, and sIndex for the short form. The two indices are initialized to point to the end of their respective strings. For each character sIndex points to, lIndex is decremented until a matching character is found. Taghva and Gilbreth [11] utilized the Longest Common Subsequence algorithm to find all possible alignments of the abbreviation to the text followed by a simple scoring rule based on matches. Chang et al. [9] also used the LCS algorithm in their machine learning method. However, state of the art alignment algorithms can not find non-acronym-type abbreviations (e.g., <Fas, CD95>), and even a little irregular acronym-type abbreviations (e.g., <CNS1, cyclophilin seven suppressor>).

Statistically based approaches always tend to extract abbreviations that appear frequently in biomedical text, and demand a large number of biomedical articles for the statistics. Zhou et al. [12] created an abbreviation database ADAM that analyzed statistical information about collocations of the type "long-form (abbreviation)" in MEDLINE. Okazaki and Ananiadou [13] built an abbreviation dictionary from the whole MEDLINE. Statistical methods can extract both acronym-type and non-acronym-type abbreviations as long as they appear frequently enough. However, they need a great deal of time and effort for the statistics, and would not find rare abbreviations even if they are only very simple acronym-type abbreviations like <DDR, DNA damage response>.

In this paper we present a systematic method for extracting biomedical abbreviations. What is crucial in this method is that a scoring strategy is utilized for classifying the abbreviations into acronym-type and non-acronym-type groups (Table 1 indicates what they mean). In the scoring strategy, the abbreviation is aligned with each of its candidate definitions using a new alignment algorithm analogous to pairwise sequence alignment [14,15], and then the definition with the largest total score is selected from the candidate definitions. If the largest total score is larger than a predefined cutoff value the abbreviation is acronym-type, or else non-acronym-type. For the acronym-type abbreviation, we use the above alignment algorithm to identify the candidate definition with the largest total score as its definition. For the non-acronym-type abbreviation, we employ a statistical method similar to Zhou et al. [12] to determine the definition. Thus, a new literature mining System MBA for extracting biomedical abbreviations is developed to recognize more abbreviations and their corresponding definitions.

**Table 1:** Acronym-type abbreviations and non-acronym-type abbreviations

abbreviations	
acronym-type	(1)regular acronym-type abbreviations: each character in the abbreviation is contained in the definition (e.g., <DC, dendritic cell>)
	(2)some irregular acronym-type ones: only one character in the abbreviation is not contained in the definition (e.g., <CNS1, cyclophilin seven suppressor>)

non-acronym-type	mainly several characters in the abbreviation are not contained in the definition (e.g., <Fas, CD95>, <5-HT, serotonin>, <Pax6, eyeless>)

## Results and discussion

**Figure 1:**
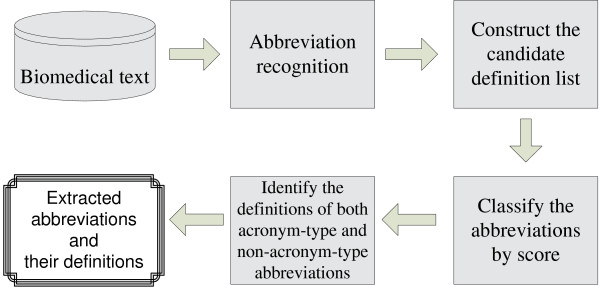
**The overall architecture of the MBA system**.

Our method consists of four steps: step 1, abbreviation recognition; step 2, construct the candidate definition list; step 3, classify the abbreviations into acronym-type and non-acronym-type groups; step 4, identify the definitions of both acronym-type and non-acronym-type abbreviations. Figure 1 shows the overall architecture of the MBA system.

### Abbreviation recognition

To obtain the abbreviations, we take into consideration the feature of an abbreviation and the syntactic cues which abbreviations occur in the contexts. The feature of an abbreviation includes: its first character is alphabetic or numeric; it contains at least one letter; its length is between 2 and 10; it contains at most two words. Park and Byrd [8] demonstrated that the syntactic cues include:

(1) long form (short form) or long form [ short form]

(2) short form (long form) or short form [ long form]

(3) short form = long form

(4) long form = short form

(5) short form, ***or ***long form

(6) long form, ***or ***short form

(7) short form...***stands/short/acronym***...long form

(8) long form, short form ***for short***

In practice, most abbreviations appear with parentheses (e.g., protein kinase C (PKC)). We use the similar method for abbreviation recognition as most researchers, and only consider pattern (1) and (2). For pattern (2), the short form is the one or two words before the left parenthesis, and the long form is just the expression inside the parentheses. For pattern (1), the short form is inside the parentheses, but the long form is not easy to be identified. Thus, we take all the parenthesized tokens, in which the strings conform to the feature of an abbreviation, to be potential abbreviations. Next we find all the possible candidate definitions for each potential abbreviation, and then identify the optimal definition.

### Construct the candidate definition list

The candidate definition appears in the same sentence as the abbreviation, and it can be searched for within a search space. The size of the search space is the sum of the maximum length of a definition (the number of the words in the definition) and the maximum offset (the longest distance of a definition from an abbreviation). In our work, the offset is ignored and we consider only definitions adjacent to the abbreviations (as most researchers do). Park and Byrd [8] analyzed about 4500 abbreviations and their definitions, and then they decided that, for relatively short abbreviations (from two to four characters), the maximum length of a definition should not be greater than twice the abbreviation length (the number of the characters in an abbreviation); for long abbreviations (five or more characters), the definition should not be longer than the abbreviation length plus 5. Thus, we refer to their work for the maximum length of a definition *DEF *of an abbreviation *ABBR*:

(1) *Max*.|*DEF*| = *min *(|*ABBR*| + 5, |*ABBR*| * 2)

where *Max*.|*DEF*| is the maximum length of a definition, and *|ABBR| *is the number of the characters in an abbreviation.

Then a candidate definition list is constructed from the search space, and the possible definition is just one item of it. The list-constructing algorithm is described in Table 2. For example, in the text "this gene is expressed in a circadian pattern in the suprachiasmatic nucleus (SCN)", |*ABBR*| = 3, *Max*.|*DEF*| = min(3+5,3*2) = 6, *SearchSpaceString *= "circadian pattern in the suprachiasmatic nucleus", *CDL *= {"circadian pattern in the suprachiasmatic nucleus", "pattern in the suprachiasmatic nucleus", "in the suprachiasmatic nucleus", "the suprachiasmatic nucleus", "suprachiasmatic nucleus", "nucleus"}.

**Table 2:** Construct the Candidate Definition List CDL>

1: Initiate an empty candidate definition list CDL;
2: Num = the number of words from the beginning of the sentence which contains the abbreviation to the left parenthesis;
3: if (Num < Max.|DEF|) { SearchSpaceString = the string from the beginning of the sentence to the left parenthesis; } else { SearchSpaceString = the string that contains Max.|DEF| words before the left parenthesis; }
4: WordNum = min (Num, Max.|DEF|);
5: for (N = 0; N < WordNum; N++) { CandidateDef = SearchSpaceString with the leftmost N words deleted; insert CandidateDef into CDL; }

### Classify the type of abbreviations

Abbreviations are classified into acronym-type and non-acronym-type abbreviations (Table 1 indicates what they mean) by scoring abbreviations and their corresponding definitions. Each time we retrieve an item from the candidate definition list, align it with the abbreviation employing our alignment algorithm, and then select the optimal definition. The score between the abbreviation and the optimal definition determines whether the abbreviation is acronym-type or not.

#### Data preprocessing

Usually a definition is abbreviated with a new addition of a special character (e.g., <Myo3/5p, Myo3p and Myo5p>), and the lowercase letter from a definition may be changed into its corresponding capital letter. Some data preprocessing steps must be taken before we identify the definition for a given abbreviation:

• delete the character that is neither alphabetic nor numeric in the abbreviation and change all capital letters in both the abbreviation and the definition into their corresponding lowercase letters.

• replace the space between words of the candidate definition with the character '\s' in order to differentiate between the space inserted in the alignment algorithm and the space between words of the candidate definition.

#### Alignment algorithm

The definition identification is a process of comparison between the abbreviation and the definition. The smallest unit of comparison is a pair of characters, one from the abbreviation, and the other from the definition. All possible comparisons are made from the smallest unit while allowing gap insertions in the abbreviation. Among the comparisons the definition with the best match is chosen as the optimal definition. The best match can be defined as the largest alignment score of characters of the definition that can be matched with those of the abbreviation. The largest alignment score can be determined by representing in a two-dimensional array, all possible pair combinations that can be constructed from the abbreviation and the definition, *A *and *D*, being compared. *A [i] *is the ith character of the abbreviation string and *D [j] *is the jth character of the definition string. *A [i] *and *D [j] *represent the rows and the columns of the two-dimensional array *SCORE*. Then the cell, *SCORE [i] [j]*, represents a pair combination that contains *A [i] *and *D [j]*.

With the above definition of *A [i]*, *D [j] *and *SCORE [i] [j]*, now what we need to do is to get the largest value of *SCORE [i] [j]*, which represents the best match. Then dynamic programming is used to compute each cell value of *SCORE*. Unlike the solutions of Needleman and Wunsch [14] and Smith and Waterman [15], we do not allow the gap insertions in the definition, so *SCORE [i] [j] *is determined by *SCORE [i] [j-1]*, *SCORE [i-1] [j-1] *and the alignment of *A [i] *and *D [j]*, and not by *SCORE [i-1] [j]*. The below is the recursion equation for computing the largest value of *SCORE [i] [j]*.

Firstly the initial value is assigned:

*SCORE [i] [j] *= 0 if *i *= 0 or *j *= 0;

Then, we have

(2) $$\begin{array}{l} SCORE[i][j]=\\ \max_{\begin{array}{c} 0<i<length(A)\\ 0<j\leq length(D) \end{array}}\{\begin{array}{l} SCORE[i-1][j-1]+w(A[i],D[j])\\ SCORE[i][j-1] \end{array} \end{array}$$

where the *w(A [i], D [j]) *is defined as:

(3) $$\begin{array}{l} w(A[i],D[j])=\\ \{\begin{array}{ll} WA, & \begin{array}{l} if\;A[i]=D[j]\;and\;D[j]\;is\;the\;first\\ character\;of\;one\;word\;in\;the\;definition; \end{array}\\ WB, & \begin{array}{l} if\;A[i]=D[j]\;and\;D[j]\;is\;not\;the\;first\\ character\;of\;one\;word\;in\;the\;definition; \end{array}\\ WC, & if\;A[i]!=D[j] \end{array} \end{array}$$

where *WA *and *WB *are positive values, *WA*>*WB*, and *WC *is a negative value.

**Figure 2:**
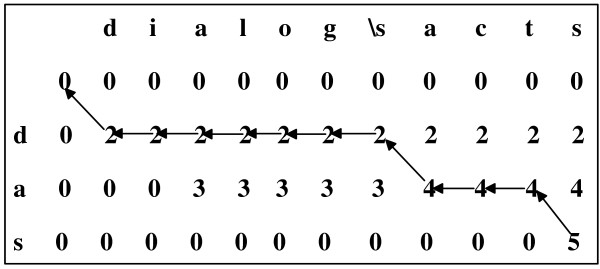
**An example for the alignment algorithm**. The definition is "Dialog Acts", and the abbreviation is "DAs". All the arrows form the best match pathway.

After the matrix *SCORE *is filled, *SCORE [length(A)] [length(D)] *is just the largest alignment score, the score of the best match. Knowing the largest alignment score is not enough, we need to get the best match pathway by traceback. The best match pathway can be obtained by beginning at the terminals of both strings (*i *= length(*A*), *j *= length(*D*)) and proceeding row by row toward the origins. The traceback algorithm checks if *SCORE [i] [j] *is obtained from *SCORE [i-1] [j-1]*. If yes, *A [i] *is identical to *D [j]*, and both *i *and *j *are decremented. If not, a space is inserted before the ith character of the abbreviation, and only *j *is decremented. The process is repeated until all cells in the matrix SCORE have been operated upon. The operation of successive summations of cell values is illustrated in Figure 2 (we assume here that *WA *= 2, *WB *= 1 and *WC *= -10).

#### Select the optimal definition

**Figure 3:**
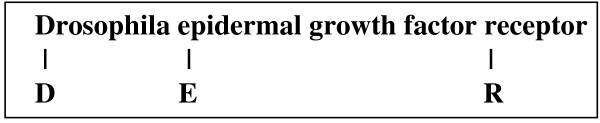
**An example for the redundant word penalty**. This is an alignment for <DER, Drosophila epidermal growth factor receptor>. In the alignment, the word "growth" in the definition is unmatched, and "factor" is also unmatched. Adjacent to each other, they are called "continuous unmatched words". The number of the continuous unmatched words is 2.

From the candidate definition list *CDL*, we can get at most *Max*.|*DEF*| candidate definitions, each of which corresponds to an alignment score provided by the alignment algorithm. Despite the alignment score, it is not enough to determine the optimal definition. For example,

1. In the text "little is known, however, about how such dialog acts (DAs) can be automatically classified in truly natural conversation", "DAs" will be recognized as an abbreviation. The true definition is "dialog acts", but "dialog acts", "such dialog acts", "how such dialog acts" and "about how such dialog acts" will have the same alignment score.

2. In the text "the mutations map across most of the Bicoid protein, with some located within the DNA-binding domain (homeodomain)", "homeodomain" will be recognized as an abbreviation wrongly. Then the alignment algorithm will select the string "with some located within the DNA-binding domain" as its definition. However, "within the DNA-binding" in the definition is unmatched in this alignment (Figure 3 illustrates what "unmatch" means). With too many unmatched words in the middle of the definition this abbreviation must be thrown away.

3. In the text "a questionnaire was sent to them as well as to 246 physicians who had residency-level teaching responsibilities but who were not named (controls)", "controls" will be recognized as an abbreviation wrongly. The alignment algorithm will select the string "physicians who had residency-level teaching responsibilities but who were not named" as its definition. However, "but who were not named" in the definition is unmatched in this alignment. With too many unmatched words in the end of the definition this abbreviation must also be thrown away.

Then we put forward a new concept: the redundant word penalty, which is defined as follows:

**Definition 1 ***The redundant word penalty is a penalty against the candidate definitions having several continuous unmatched words*.

The penalty depends on the number of the continuous unmatched words in the candidate definition (Figure 3). If the number is small, the penalty is low, otherwise the penalty is high. One unmatch word often appears in true definitions, for example, for the pair <FMDV, foot and mouth disease virus>, there is only one unmatched word "and" in the definition. The penalty should be very low in this case. Based on the analysis, the redundant word penalty (RWP) is divided into the beginning word penalty (*BP*, a low penalty) and the extended word penalty (*EP*, a high penalty). In *N *continuous unmatched words, the first *C *words are given a penalty score, *BP *for each word, and the other *N-C *words are given another penalty score, *EP *for each word. Thus, the equation of *RWP *is as follows:

(4) *RWP *= C * BP + EP * (*N *- *C*)

There are three cases (corresponding to the above three examples respectively) that the redundant word penalty occurs:

1. The first character of the abbreviation does not match the first word of the candidate definition (*RWP1*).

2. Two adjacent characters in the abbreviation match two separated words in the candidate definition separately (*RWP2*).

3. The last character of the abbreviation does not match the last word of the candidate definition (*RWP3*).

For example, for the alignment <DER, Drosophila epidermal growth factor receptor> in Figure 3, *RWP1*, *RWP2 *and *RWP3 *are computed as follows:

1. *RWP1 *= 0, because the first character "D" in the abbreviation matches the first word "Drosophila" of the definition.

2. For *RWP2*, any two adjacent characters in the abbreviation must be considered. "D" and "E" match two adjacent words "Drosophila" and "epidermal" respectively, so *RWP2("DE") *= 0; "E" and "R" match two separated words "epidermal" and "receptor" respectively, and the number of the continuous unmatched words is 2, so *RWP2("ER") *= C *BP + EP*(2-*C*). In sum, *RWP2 *= *RWP2("DE") *+ *RWP2("ER")*.

3. *RWP3 *= 0, because the last character "R" in the abbreviation matches the last word "receptor" in the definition.

The three cases may appear in the same alignment, so the total redundant word penalty (*TotalRWP*) is:

(5) *TotalRWP *= *RWP1 *+ *RWP2 *+ *RWP3*

Then for each alignment, we have

(6) *total score *= *alignment score - TotalRWP*

At last the optimal definition can be selected from the candidate definition list by selecting the largest *total score*. If the *total score *of the optimal definition is larger than the predefined cutoff score, the abbreviation is acronym-type, otherwise non-acronym-type.

Here, the *w(A [i], D [j])*, the redundant word penalty, the cutoff score and the variable *C *(in equation 4) need to be adjusted. To assist in assigning the optimal value for the above, a publicly available tagged corpus, the Medstract gold standard DEVELOPMENT corpus is used. We first gave each of the above parameters an initial value, and then for each one we examined how the total score for the optimal definition were distributed when we changed the value keeping the other parameters unchanged. To truly differentiate acronym-type and non-acronym-type abbreviations, the values are set as follows:

• *WA *2

• *WB *1

• *WC *-7

• *BP *0.5

• *EP *4.5

• *C *1.0

• the cutoff score 0.5

### Identify the definitions of both acronym-type and non-acronym-type abbreviations

We could separately identify the definitions of acronym-type and non-acronym-type abbreviations after the abbreviations are classified. For the acronym-type abbreviation, we use the above alignment algorithm to traceback for the definition with the largest total score, and thus the definition is identified. For the non-acronym-type abbreviation, we use a statistical method similar to that of Zhou et al. [12].

Our statistical method is based on PubMed (a service of the U.S. National Library of Medicine that includes over 17 million citations from MEDLINE and other life science journals for biomedical articles back to the 1950s), in which we count the number of articles that contain both the candidate definition and the abbreviation. Zhou et al. [12] got statistical information of "candidate definition (abbreviation)" including a pair of parentheses, whereas the abbreviation and the definition may not be separated by parentheses or not appear in the same sentence but the same article. It is too complex to consider all the syntactic clues which abbreviations occur in the contexts in abbreviation recognition, but the statistical method need not consider the specific syntactic clues and only care about whether the definition and the abbreviation co-occur in the same article. We assume that the abbreviation is "abbr", and the candidate definition with i words is: "w_*i*_...w_2_w_1_". Thus, we count the number of articles in PubMed for each step in the progression "abbr" → "w_1 _AND abbr" → "w_2_w_1 _AND abbr" →...→ "w_*i*_...w_2_w_1 _AND abbr", and then assign each candidate definition/abbreviation pair a score that will be used to identify the candidate definition for a given abbreviation. The same score apr_*i*_, the adjusted proportion of the raw proportion pr_*i*_, is used for identifying the candidate definition as Zhou et al. [12]:

(7) $$pr_{i}=\frac{count["w_{i}w_{i-1}...w_{2}w_{1}"AND"abbr"]-1}{count["w_{i-1}...w_{2}w_{1}"AND"abbr"]},i\geq 1$$

(8) $$apr_{i}=pr_{i}-2*\sqrt{\frac{pr_{i}*(1-pr_{i})}{count["w_{i-1}...w_{2}w_{1}"AND"abbr"]}},i\geq 1$$

where count ["*w*_*i*_*w*_*i*-1_...*w*_2_*w*_1_" AND "abbr"] is the number of articles in which both the w_*i*_w_*i*-1_...w_2_w_1 _and the abbreviation occur. In equation 8 it requires that *pr*_*i *_≥ 0, so count ["w_*i*_w_*i*-1_...w_2_w_1 _"AND "abbr"] must be larger than zero in equation 7. If count ["w_*i*_*w*_*i*-1_...w_2_w_1_" AND "abbr"] is equal to zero, we assign it the value 1. For example, in the text "a lupus-like murine model of CD95 (Fas)", the adjusted proportion apr_*i *_is computed as:

$$pr_{1}=\frac{8181-1}{17318}=0.4723$$

$$apr_{1}=0.4723-2*\sqrt{\frac{0.4723*(1-0.4723)}{17318}}=0.4647$$

$$pr_{2}=\frac{1-1}{8181}=0$$

*apr*_2 _= 0

The apr value drops significantly from "CD95 (Fas)" to "of CD95 (Fas)", and thus "CD95" is determined as the definition of "Fas". In order to determine the cutoff score for the adjusted proportion, we extracted all the abbreviations and their candidate definitions in the Medstract Gold Standard DEVELOPMENT Corpus which contains 126 <"short form", "long form"> pairs, and then computed the apr value for all the candidate definitions. At last we found that 0.05 was still fit for the cutoff score as in Zhou et al. [12], which means that if the apr value drops below 0.05 when the candidate definition w_*i*_w_*i*-1_...w_2_w_1 _is expanded to w_*i*+1_w_*i*_w_*i*-1_...w_2_w_1_, w_*i*_w_*i*-1_...w_2_w_1 _is identified as the definition of the abbreviation. Here, we also do not apply the cut off criteria to apr_1 _and instead require that count ["w_1_" AND" abbr"] ≥ 10. In summary, given the abbreviation "abbr" and the candidate definition "*w*_*k*_...*w*_2_*w*_1_", *w*_1 _is a candidate definition identified by

$$\{\begin{array}{l} count["w_{1}"AND"abbr"]\geq 10\\ apr_{2}<0.05 \end{array}$$

*w*_*k*_...*w*_2_*w*_1 _is a candidate definition identified by

$$\{\begin{array}{l} count["w_{1}"AND"abbr"]\geq 10\\ apr_{i}\underset{2\leq i\leq k}{\geq }0.05\\ apr_{k+1}<0.05 \end{array}$$

In the above method, several candidate definitions may exist for a given abbreviation. The change of apr is also used to get rid of redundant candidate definitions as in the method of Zhou et al. [12]: given two candidate definitions of the same abbreviation, *w*_*m*_...*w*_2_*w*_1 _and *w*_*n*_...*w*_*m*_...*w*_2_*w*_1_(*m*<*n*), then

(9) $$\Delta apr=\frac{apr_{m}-apr_{n}}{apr_{m}}$$

if Δapr ≥ 0.18, remove *w*_*n*_...*w*_*m*_...*w*_2_*w*_1_, otherwise remove *w*_*m*_...*w*_2_*w*_1_. If the first word of the candidate definition is in the PubMed stopword list , the first word is removed from the candidate definition. Finally a filtering rule is adopted: the length ratio (the number of alphanumeric characters of the definition vs the abbreviation) should be larger than 1. In the paper of Zhou et al. [12], the length ratio must be either equal to or larger than 2.5 because 95% of the single-word abbreviations in the Stanford Abbreviation Database have length ratio ≥ 2.5, but our statistical method is only for non-acronym-type abbreviations. There are many non-acronym-type abbreviations such as <Fas, CD95>, <Pax6, eyeless> and so on, and their length ration is neither equal to nor larger than 2.5, but generally larger than 1.

### Evaluation

#### Evaluation on the Medstract Gold Standard Corpus

To evaluate the MBA system, we have run it against a publicly available tagged corpus, the Medstract Gold Standard Corpus, which is composed of the DEVELOPMENT corpus with 126 tagged <short form, long form> pairs and the EVALUATION corpus with 168 tagged pairs. The DEVELOPMENT corpus has been used for determining some values before, and the EVALUATION corpus is used for comparing the MBA system with three popular downloadable algorithms:

• the Chang et al. [9] algorithm (obtained from ) at the three cutoff scores: 0.03, 0.14 and 0.88.

• the SLICE algorithm [5] (obtained from ).

• the S&H algorithm [10] (obtained from ).

Our result is strictly based on the corpus without corrections, and the extracted pairs must match the tagged ones exactly. MBA identified 162 <short form, long form> pairs in the result. Out of these, 147 pairs were correct, resulting in a recall of 88% and a precision of 91%. Table 3 indicates the result of that comparison with other algorithms on the gold-standard corpus.

**Table 3:** Comparing with other algorithms on the gold-standard EVALUATION corpus

	Precision	Recall	F-measure
Chang (score = 0.88)	93%	23%	0.37

Chang (score = 0.14)	89%	76%	0.82

Chang (score = 0.03)	87%	81%	0.84

ALICE	90%	77%	0.83

S & H	91%	77%	0.83

MBA	91%	88%	0.89

In our result fifteen pairs were incorrect: nine pairs were only partially matched (Table 4); the rest pairs might be <short form, long form> pairs, but not biomedical items (un-tagged in the corpus):

**Table 4:** The partially matched definitions by MBA

abbr.	true definition	extracted definition
TFIIB	general transcription factor IIB	transcription factor IIB

Pol I	RNA polymerase I	polymerase I

Pol II	RNA polymerase II	polymerase II

VHL	multiprotein von Hippel-Lindau	von Hippel-Lindau

PKA	cAMP-dependent protein kinase A	protein kinase A

Hh	protein Hedgehog	Hedgehog

Ci	transcription factor Cubitus interruptus	Cubitus interruptus

Fu	protein kinase Fused	Fused

O-glycans	serine/threonine-linked oligosaccharides	oligosaccharides

• lethal of scute (l'sc)

• basic helix-loop-helix (bHLH)

• primary ethylene response element (PERE)

• Ca2+-sensing receptor (CaSR)

• intermediate neuroblasts defective (ind)

• eliminates an AP180 homolog (LAP)

The system MBA missed twenty-one pairs: nine of them were only partially matched, that is to say, the true definition includes an additional word, for example, "RNA polymerase I (Pol I)", MBA missed the word "RNA"; three non-acronym-type abbreviations were not found because of the insufficient statistical information; for the other nine pairs, the definition and abbreviation were not separated by parentheses. Since nine <short form, long form> pairs are not separated by parentheses, they will be ignored by the abbreviation recognition algorithm. If we do not consider more syntactic cues for abbreviation recognition, MBA can only achieve the highest recall of 95% even if the alignment algorithm and the statistical method are perfect.

Moreover, we also analyzed the abbreviation database ADAM [12] based on the gold standard EVALUATION corpus. Firstly, we extracted all of the 168 tagged abbreviations and their corresponding definitions; Secondly, manually input the abbreviations one by one into the form of the webpage  and then searched the database to check if their corresponding definitions were in the list of "Long-forms and variants". In this way, only 87 abbreviations and their definitions were found in ADAM, resulting in a recall of 52%. This shows that the statistical method can not recognize rare abbreviations, and it is not effective to employ only the statistical method. If our alignment algorithm was solely run on the corpus, it identified 153 pairs. Out of these, 139 pairs were correct, resulting in a recall of 83% and a precision of 91%. Through analyzing the result, we found that many non-acronym-type abbreviations were discarded. So it is necessary for the alignment algorithm to explore the statistical method as described in the paper of Torii et al. [16].

#### Error analysis

The Medstract Gold Standard Corpus is not large enough for error analysis, so the top 1500 abstracts were selected from the results of a query on the term "protein" in PubMed. In the larger corpus [17], we ran the MBA system and then investigated how many false <short form, long form> pairs in the result.

The MBA system identified 2491 <short form, long form> pairs in total, and 119 errors were found, giving an error rate of 4.78%. There were three types of errors as follows:

1. There were 22 errors in the phase of abbreviation recognition. Twenty-two parenthesized tokens were wrongly recognized as abbreviations (e.g., cis-diamineplatinum (II)).

2. Some errors (41/119) occurred when the system identified the definitions of the acronym-type abbreviations. The system got either a longer string or a shorter string than the true definition for an abbreviation. Fox example, for the definition "regulatory T cells" of the abbreviation "Tregs", the system wrongly identified "that regulatory T cells" as its definition.

3. Some errors (56/119) occurred when the system identified the definitions of the non-acronym-type abbreviations. For example, "effective half-maximal concentration" was the definition of "EC(50)", but the system wrongly identified "concentration" as the definition.

## Conclusion

In this paper, we develop a systematic method for extracting biomedical abbreviations. It consists of four steps mainly: step 1, abbreviation recognition; step 2, construct the candidate definition list; step 3, classify the abbreviations into acronym-type and non-acronym-type groups; step 4, separately identify the definitions of acronym-type and non-acronym-type abbreviations: text alignment algorithm for the former, statistical method for the latter. Our evaluation demonstrates that the MBA system performs better than the others. It can identify the definition of not only acronym-type abbreviations including a little irregular acronym-type abbreviation(e.g., <CNS1, cyclophilin seven suppressor>), but also non-acronym-type abbreviations.

The MBA system needs a few improvements, although it is good at extracting both acronym-type and non-acronym-type abbreviations. In our study we use a simple method to select the best values for several parameters. At present there are many optimizing methods, such as Genetic Algorithm, Simulated Annealing Algorithm and so on. We have been trying to optimize the parameters with these methods, and this is the topic of our current research. Besides the needed improvement in parameter optimization, the statistical method costs a lot of time in the MBA system, and we need reduce the time cost by either narrowing the searching range or paralleling our algorithm. This is another topic of our current research. Our future work is to set up a biomedical abbreviation server, in which we will consider more syntactic clues in the contexts for better results.

In conclusion, a literature mining system MBA is developed and applied to extract biomedical abbreviations. MBA could find both acronym-type and non-acronym-type abbreviations effectively. The systematic method can also be used in the general text, or applied in other research areas.

## Methods

### data sources

The Medstract Gold Standard Corpus [16] and a larger corpus [16] are used in this paper. The gold standard corpus is just a publicly available tagged corpus, and it is composed of DEVELOPMENT corpus and EVALUATION corpus. The DEVELOPMENT corpus contains 126 <short form, long form> pairs, and the EVALUATION corpus contains 168 pairs. The larger corpus contains 1500 abstracts which were selected from the results of a query on the term "protein" in PubMed.

### Evaluation of the method

We use the harmonic mean (F-measure) of precision (accuracy) and recall (coverage) that are commonly used in the field to evaluate our results. The precision measures the number of correct <short form, long form> pairs in the answer file over the total number of the pairs in the answer file, and the recall measures the number of correct pairs in the answer file over the total number in the key file. With "TP" labeling true positives, "FP" the false positives and "FN" the false negatives, the measures are:

(10) $$\begin{array}{l} \text{Precision}=\frac{TP}{TP+FP}\\ \text{Recall}=\frac{TP}{TP+FN}\\ \text{F-measure}=\frac{2*Precision*Recall}{Precision+Recall} \end{array}$$

## Authors' contributions

ZW designed and implemented the MBA system, analyzed the results and drafted the manuscript. YX partly designed the alignment algorithm, analyzed the results and revised the manuscript. YL, YX and YZ coordinated the project and revised the final manuscript. All authors read and approved the final manuscript.
